# Premeal insulin administration lowers postprandial blood glucose and increases myocardial microvascular blood flow in people with type 1 diabetes: a randomised, crossover clinical trial

**DOI:** 10.1007/s00125-026-06806-2

**Published:** 2026-07-11

**Authors:** William B. Horton, Kara C. Anderson, Kaitlin M. Love, Linda A. Jahn, Lee M. Hartline, James T. Patrie, Jia Liu, Michael D. Solga, Wilhelmina J. Awan, Archana Thakur, Dana L. Schalk, Mason G. Becker, Catherine E. Kuzas, Kevin W. Aylor, Eugene J. Barrett

**Affiliations:** 1 Division of Endocrinology and Metabolism, Department of Medicine, University of Virginia School of Medicine, Charlottesville, VA USA; 2 University of Virginia Center for Diabetes Technology, University of Virginia School of Medicine, Charlottesville, VA USA; 3 AdventHealth Translational Research Institute, Orlando, FL USA; 4 Division of Biostatistics, Department of Public Health Sciences, University of Virginia School of Medicine, Charlottesville, VA USA; 5 Flow Cytometry Core, University of Virginia School of Medicine, Charlottesville, VA USA; 6 Center for Human Therapeutics cGMP Facility, University of Virginia School of Medicine, Charlottesville, VA USA; 7 Department of Pharmacology, University of Virginia School of Medicine, Charlottesville, VA USA

**Keywords:** Carbohydrate metabolism, Cardiac complications, Endothelium, Insulin action, Insulin therapy, Microvascular disease

## Abstract

**Aims/hypothesis:**

We aimed to evaluate whether prandial insulin timing affects vascular function in people with type 1 diabetes. Our hypothesis was that premeal insulin administration would lead to greater myocardial microvascular blood flow (MBF) via blunting postprandial hyperglycaemia.

**Methods:**

People with type 1 diabetes between 18 and 35 years of age with BMI <30 kg/m^2^ underwent two protocols with a 1:1 randomised crossover design wherein prandial insulin was injected either 15 min before or 15 min after meal intake began. To provide a physiological comparison, age-, sex- and BMI-matched control participants completed one study where they consumed the same meal but received no exogenous insulin. Glucose, insulin, vascular function (including ultrasound measures of myocardial and skeletal muscle microvascular perfusion, aortic stiffness, brachial artery endothelial function) and biomarkers of systemic inflammation and endothelial dysfunction were assessed at baseline and then 2 h after meal ingestion within each protocol. The primary outcome was change in myocardial MBF within each protocol. Study personnel assessing outcomes were masked to group assignment.

**Results:**

Eighteen people with type 1 diabetes and 18 matched control participants were analysed within each protocol. Glucose area under the curve was significantly greater (*p*=0.015) in the postmeal insulin study compared with the premeal insulin study in participants with type 1 diabetes. Myocardial microvascular flow velocity significantly increased (*p*=0.031) with premeal insulin administration in people with type 1 diabetes and this consequently led to greater myocardial MBF (*p*=0.044). There were no changes in myocardial MBF within the other protocols. Changes in vital signs were similar between all protocols.

**Conclusions/interpretation:**

Appropriately timed premeal insulin led to lower postprandial blood glucose along with increased myocardial MBF in people with type 1 diabetes. Further work is needed to determine the underlying aetiology of these changes.

**Trial registration:**

ClinicalTrials.gov NCT04730882

**Graphical Abstract:**

**Figure:**
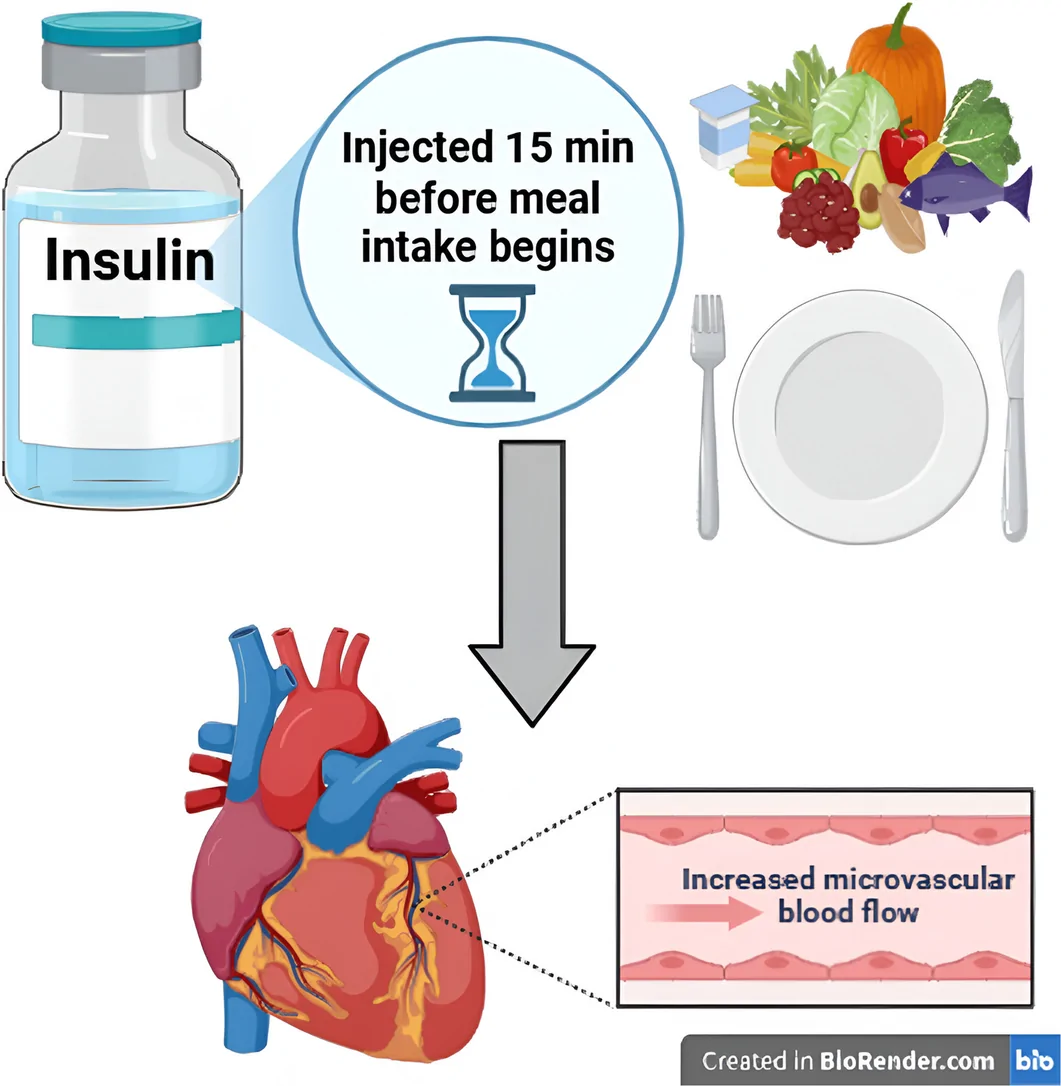

**Supplementary Information:**

The online version contains peer-reviewed but unedited supplementary material available at https://doi.org/10.1007/s00125-026-06806-2.

**Figure:**
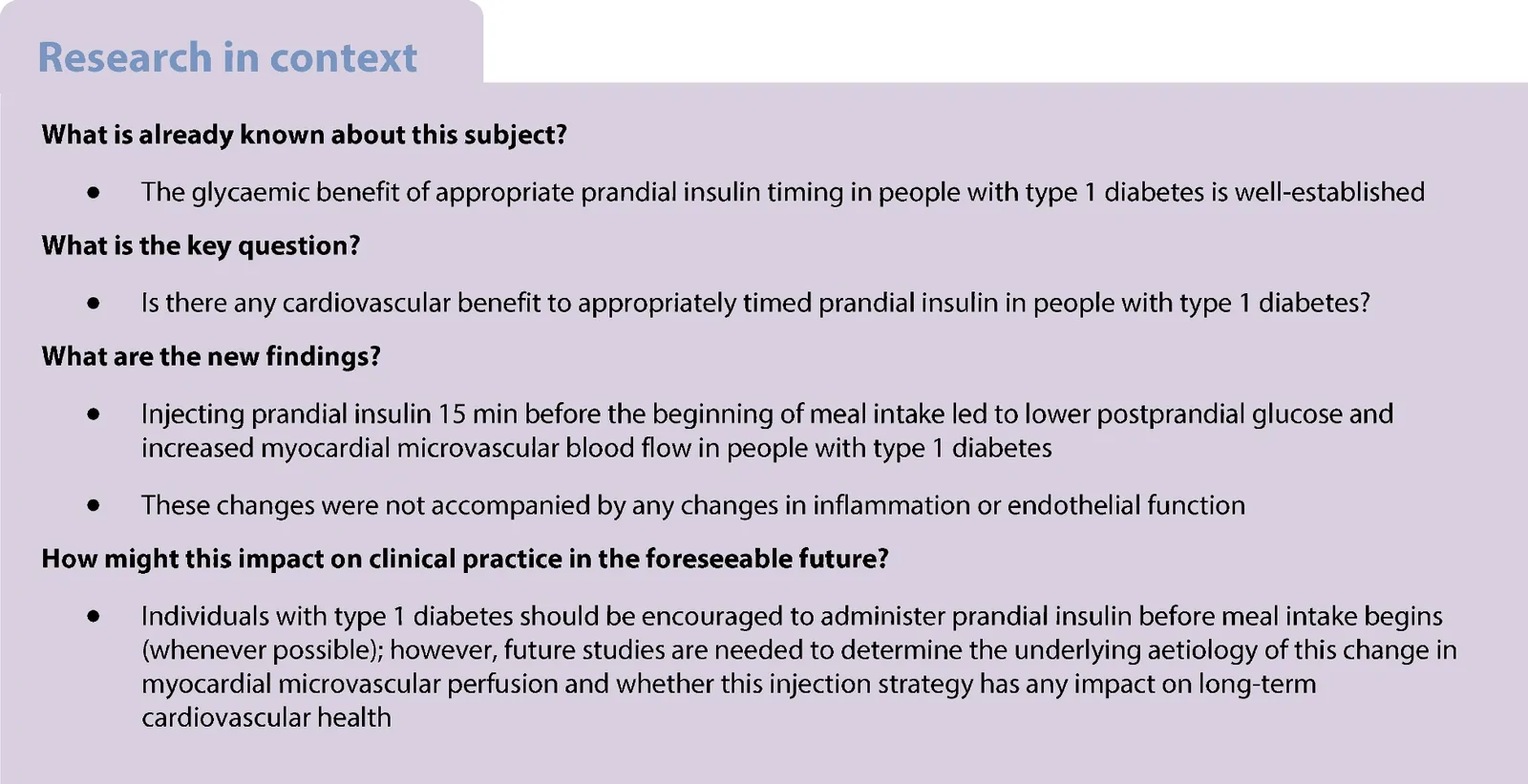

## Introduction

The importance of optimal glycaemic control in preventing chronic macrovascular complications in people with type 1 diabetes is well-established [1–3]; however, recent real-world observational analyses have shown that many (ranging anywhere from 42.3% to 79%) adults with type 1 diabetes do not achieve recommended glycaemic targets [4–6]. For example, even in the setting of closed-loop automated insulin delivery (AID), anywhere from 34% to 69% of participants with type 1 diabetes did not achieve recommended glycaemic targets in several randomised controlled clinical trials [7–10]. This inability to achieve optimal control is multifaceted; however, postprandial hyperglycaemia is a key contributor [11–14]. Concerningly, various studies have shown that postprandial hyperglycaemia is a significant, independent risk factor for cardiovascular disease in people with diabetes and that it may be more predictive of cardiovascular events than either fasting glucose or mean HbA_1c_ [15–17].

While timing of prandial insulin is ideally tailored to individual circumstances, it is generally recommended that rapid-acting insulin be administered ~15–20 min before meal intake begins to minimise postprandial hyperglycaemia [12]. Notably, people with type 1 diabetes who routinely follow this recommendation achieve better HbA_1c_ values over time [18]. Despite the known benefits of appropriate prandial insulin timing on glycaemic control, the cardiovascular impact of this intervention has, to our knowledge, not been investigated in people with type 1 diabetes. Thus, we designed the current study to evaluate whether prandial insulin timing affects vascular function in people with type 1 diabetes. Our hypothesis was that premeal insulin administration would lead to greater myocardial microvascular blood flow (MBF) via blunting postprandial hyperglycaemia and stimulating endothelial function in people with type 1 diabetes.

## Methods

### Study design, recruitment strategies and study population

There was no patient, public or funder involvement in the design, conduct and reporting of this trial. Recruitment was achieved by community advertisement (e.g. flyers and digital advertisements to the public) and direct advertisement to local healthcare clinics both within and outside the University of Virginia (UVA) Health System. We recruited two separate cohorts for this study: one consisting of people with type 1 diabetes and a separate cohort of age- (±2 years), sex- (1:1 and determined by self-report) and BMI- (±1 kg/m^2^) matched control participants. We followed the Consolidated Standards of Reporting Trials (CONSORT) guidelines for reporting this randomised crossover clinical trial. Please see electronic supplementary material (ESM) Table 1 for a completed CONSORT checklist.

### Clinical assessment and initial screening

All screening visits and clinical studies were conducted at the UVA Clinical Research Unit (CRU). Each participant provided written informed consent at their initial visit prior to being carefully screened to verify inclusion/exclusion criteria. Screening included a detailed medical history and physical examination along with fasting assessment of complete blood count, comprehensive metabolic panel, lipid panel, plasma glucose and serum pregnancy test (i.e. hCG). People with type 1 diabetes met inclusion criteria if they were ≥18 and ≤35 years old, had BMI of 18–30 kg/m^2^ and had type 1 diabetes (based on clinical history and World Health Organization diagnostic criteria for diabetes) for ≥1 year. Key exclusion criteria included blood pressure (BP) ≥140/90 mmHg, pulse oximetry <90%, LDL-cholesterol ≥4.14 mmol/l (160 mg/dl) or HbA_1c_ ≥75 mmol/mol (9%) at time of screening; current smokers or those who quit smoking <2 years ago; use of vasoactive medications; screening electrocardiogram findings indicative of arrhythmia, sinus node disease or ischaemic heart disease; or current pregnancy. Control participants had to meet the same inclusion/exclusion criteria but could not have diabetes based on World Health Organization diagnostic criteria. Please see ESM Table 2 for a complete list of inclusion/exclusion criteria and ESM Fig. 1 for the CONSORT flowchart.

### Experimental protocols

**Fig. 1:**
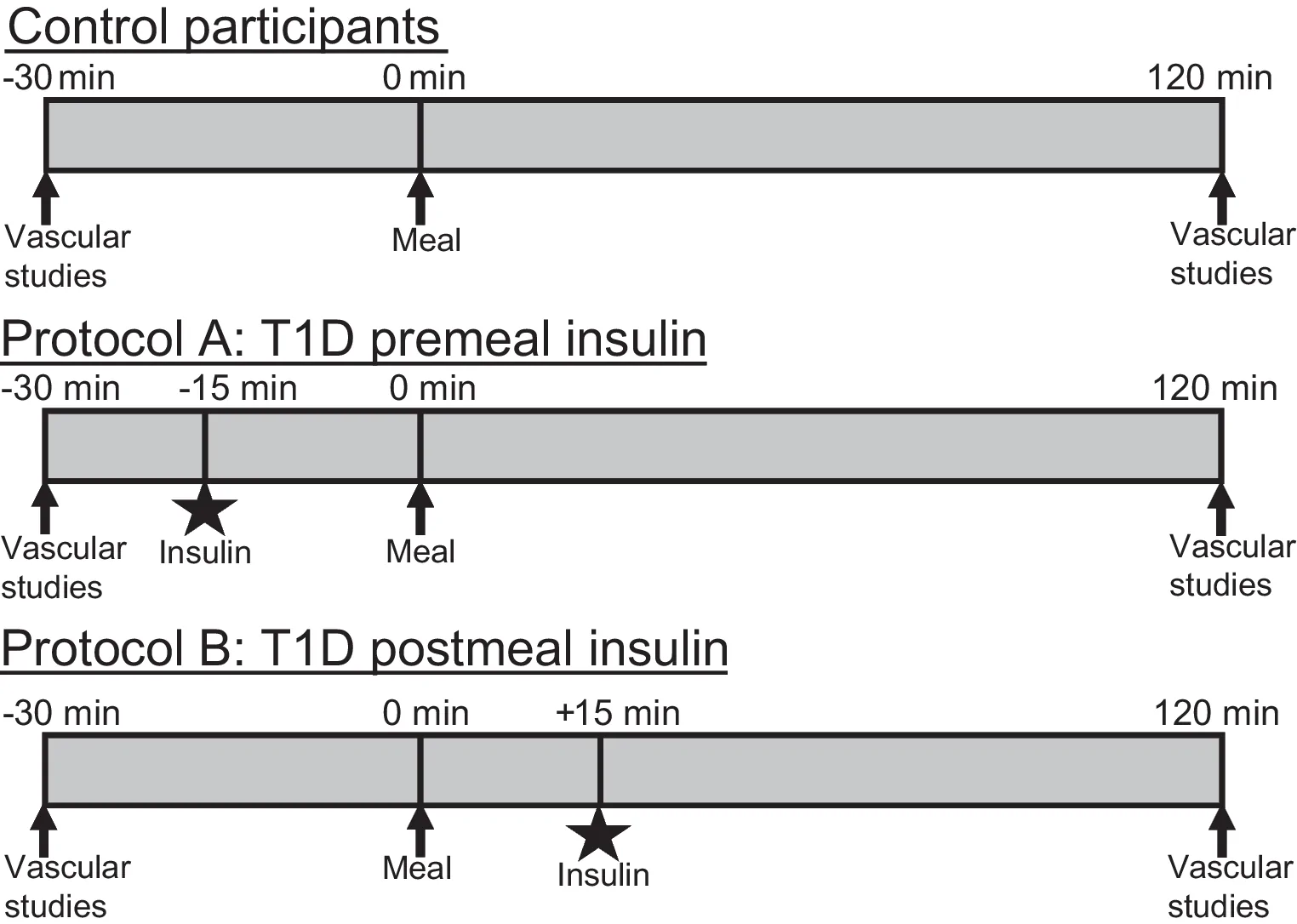
Experimental protocols. T1D, type 1 diabetes

All study protocols received institutional review board approval and were conducted in accordance with the Declaration of Helsinki (2024 revision). All participants were advised to abstain from caffeine and alcohol intake and avoid physical activity for 24 h prior to each study date. Participants then reported to the UVA CRU on the morning of the study following an overnight fast. Glucose data were reviewed with all type 1 diabetes participants to ensure they did not experience any severe hypo- (i.e. glucose <3.1 mmol/l or 55 mg/dl) or hyperglycaemia (glucose >13.9 mmol/l or 250 mg/dl) in the 24 h prior to study. Our goal was to have participants with type 1 diabetes arrive on the study date with a blood glucose within the prespecified target range (i.e. 5.6–8.3 mmol/l or 100–150 mg/dl) and thus we conducted basal testing with subsequent dose optimisation (if needed) in the 3 days leading up to the study. If participants with type 1 diabetes arrived on the study date with a blood glucose outside the target range, they received either a slow glucose infusion (if glucose <5.6 mmol/l or 100 mg/dl) or a low-dose insulin infusion (if glucose >8.3 mmol/l or 150 mg/dl) for up to 2 h to bring glucose to target range before study initiation. Baseline values of biochemical biomarkers and vascular function were then obtained. Following this, all participants consumed a standardised liquid mixed meal (41.84 kJ/kg body weight) and vascular measurements along with plasma biomarkers were collected 120 min later (Fig. 1). Participants with type 1 diabetes underwent two protocols (separated by ≥1 but ≤4 weeks) with a 1:1 randomised crossover design via a randomisation sequence generated by the study biostatistician: (A) prandial insulin lispro was injected 15 min before meal intake began (per current recommendations [12]); and (B) prandial insulin lispro was injected 15 min after meal intake began (to allow postprandial hyperglycaemia and mimic a common real-world scenario [12, 19]). Personnel who assigned participants to specified interventions had access to the randomisation sequence. An identical prandial insulin dose was administered by study personnel within each protocol based on the specific insulin:carbohydrate value of each participant; however, we did not try to optimise their insulin:carbohydrate values before study initiation so that we could mimic each participant’s real-world clinical results. Participants on multiple daily injections were required to use the same basal insulin dose on each study date to limit variation in basal insulin levels. Similarly, participants on closed-loop AID were required to switch their pump over to manual mode 3 h prior to study initiation to limit variation in basal insulin levels between study dates. The liquid meal contained 58%, 27% and 15% of calories from carbohydrates, fat and protein, respectively. Plasma insulin and glucose were measured every 30 min throughout the study. To provide a physiological comparison, matched control participants completed one study where they consumed the same meal but received no exogenous insulin. There were no changes to the experimental protocols after the study commenced.

### Assessment of vital signs

Clinical haemodynamic assessments were collected on each study date at baseline and then 2 h after meal ingestion. Systolic blood pressure (SBP), diastolic blood pressure (DBP) and heart rate (HR) were obtained with a GE Carescape VC150 vital signs monitor (GE Healthcare Technologies; Chicago, IL). Rate pressure product (RPP) was calculated as the product of SBP and HR (i.e. SBP × HR).

### Assessment of vascular function

#### Carotid–femoral pulse wave velocity

We followed recommendations from the American Heart Association Scientific Statement to assess and report carotid–femoral pulse wave velocity (cfPWV) [20]. To minimise the effects of sympathetic activity on cfPWV, participants laid supine in a temperature-controlled room for at least 15 min prior to measurement. We measured the distance from the suprasternal notch to the carotid pulse and from the suprasternal notch to the femoral pulse on the same side. All cfPWV measurements were conducted with a SphygmoCor CVMS tonometer (Atcor by CardieX; NSW, Australia). For each measure, 10 s of carotid and 10 s of femoral arterial waveforms were recorded. cfPWV measures were made in duplicate and the mean value was reported. cfPWV intraobserver reliability was also assessed by having the operator record three serial cfPWV measurements on the same participant over a 4 h period. The coefficient of variation for cfPWV was 3.63%, indicating good intraobserver reliability [21].

#### Brachial artery flow velocity and flow-mediated dilation

We also followed evidence-based recommendations [22] for the assessment and reporting of brachial artery flow velocity and flow-mediated dilation (FMD) in humans. A high-resolution cardiovascular ultrasound (EPIQ 7; Philips Medical Systems; Andover, MA) instrument with a linear transducer (L12–5) was used to evaluate brachial artery blood flow and FMD. Participants were instructed to lay supine with left forearm extended. The location of the probe was marked at baseline to allow for consistent probe placement for repeated measures. A manual BP cuff was placed distally from the antecubital fold and was inflated to 250 mmHg for forearm occlusion for 5 min. Brachial artery blood velocity was obtained during the first 20 s after cuff deflation and diameter obtained every 10 s from 30 to 120 s. Baseline and peak diameter values were obtained for the FMD calculation, and peak velocities were automatically calculated by the ultrasound machine and recorded. FMD images were analysed using Brachial Analyzer version 6.15.18 (Medical Imaging Applications; Coralville, IA) edge detection software by study personnel blinded to participant and protocol. We used 5 s time bins to identify peak FMD. The interclass correlation coefficient for our lab operator is 0.91. Post-occlusion peak shear rate was calculated using the following formula: 4 × (maximal velocity/brachial artery diameter) at time of peak velocity [23, 24].

#### Contrast-enhanced ultrasound

After assessment of FMD, each participant rested for 15 min before cardiac and skeletal muscle MBF were assessed with an Epiq 7 ultrasound system during infusion of lipid-shelled octafluoropropane microbubbles (Definity; Lantheus Medical Imaging; North Billerica, MA). Microbubbles were diluted to 15 ml total volume in normal saline (154 mmol/l NaCl) and infused at a constant rate of 1.5 ml/min. Once the systemic concentration of microbubbles reached steady state (~4 min), a 5-frame high-power (mechanical index [MI]=0.88) sequence was applied to destroy microbubbles in the specific anatomical imaging sector through inertial cavitation, after which there was an immediate return to real-time, continuous imaging with MI <0.1. Electrocardiographically triggered end-systolic frames were acquired to assess contrast replenishment in the myocardium. Imaging gain, depth and focus were optimised for each participant and kept constant throughout the study. We oriented the ultrasound probe such that it optimised view of the interventricular septum during baseline testing and then marked probe location such that it was in the same location for end-of-study assessment of the myocardium. For assessment of skeletal muscle, transverse images of the left proximal forearm (approximately 5 cm distal to the antecubital fossa) were obtained. Four 20 s replenishment curves for myocardium and four 30 s replenishment curves for left forearm flexor skeletal muscle were acquired at both baseline and end of study. For quantitative analysis, replenishment curves were analysed with Q-Lab version 5.5.4 (Philips Research; Cambridge, MA) by study personnel that were blinded to participant and protocol. The first post-destructive frame was digitally subtracted from all subsequent frames and background-subtracted time-intensity data were fit to the function: *y*=*A*(1−e^−*βt*^), where *y* is signal intensity at time *t*, *A* is the plateau intensity reflecting relative microvascular blood volume (MBV) and *β* is the rate constant reflecting microvascular flow velocity (MFV). MBF was quantified by the product of MBV and MFV [25].

### Biochemical analyses

Complete blood count, comprehensive metabolic panel, lipid panel, fasting plasma glucose and serum pregnancy tests were assayed at the UVA Clinical Chemistry Laboratory. Plasma glucose was measured with a YSI 2700 Biochemistry Analyzer (Yellow Springs Instrument Company; Yellow Springs, OH) in duplicate throughout each protocol with values subsequently averaged at each 30 min interval. Given that our study evaluated people with type 1 diabetes and matched healthy control individuals, plasma insulin was measured at the same time points with the Mercodia Iso-Insulin ELISA (Mercodia; Uppsala, Sweden) so that total (i.e. both endogenous and exogenous) insulin could be quantified. Assays were read on a Synergy 2 microplate reader (BioTek; Winooski, VT).

Plasma samples of cardiovascular (including non-esterified fatty acids [NEFA], inflammatory markers and endothelial dysfunction markers) and hormonal biomarkers were collected at baseline and 120 min after meal ingestion (Fig. 1). NEFA were measured using the Wako HR series NEFA-HR (2) assay kit (Fujifilm Healthcare Americas Corporation; Lexington, MA). Cardiovascular and hormonal biomarkers were measured using Sigma Millipore Milliplex (MilliporeSigma; Burlington, MA) assay kits: HCYTA-60K-02 (interleukin-6 [IL-6] and tumour necrosis factor-ɑ [TNF-α]), HCVD2MAG-67K-01 (intercellular adhesion molecule-1 [ICAM-1]), HCVD3MAG-67K-01 (C-reactive protein [CRP]), HCVD4MAG-67K-01 (E-selectin) and MSHMAG-21K-01 (progesterone). These assays were performed according to the manufacturer’s protocol, acquired on a Luminex xMAP Intelliflex platform and analysed using Milliplex Analyst 5.1 software version 5.1.0.0 (MilliporeSigma; Burlington, MA).

### Statistics

#### Outcomes

The primary outcome for each protocol was change in myocardial MBF. Secondary outcomes included changes in vital signs, skeletal muscle MBF, FMD, cfPWV, IL-6, TNF-ɑ, CRP, ICAM-1 and E-selectin.

#### Sample size

An a priori sample size calculation with an assumed two-sided type 1 error rate of 0.05 indicated that 16 participants with type 1 diabetes would need to complete the randomised crossover protocol in order to have at least 0.80 power to detect a minimal difference in mean myocardial MBF values of 1.3 video intensity (VI)/s. This calculation was based on the observed values in standard deviation and detected differences in myocardial MBF from a prior study of people with type 2 diabetes by Scognamiglio et al [26]. We anticipated a 20% dropout rate and initially planned to enrol up to 20 participants with type 1 diabetes; however, there were no dropouts so we discontinued enrolment after 18 people with type 1 diabetes completed the randomised protocol. As noted above, we also enrolled age-, sex- and BMI-matched healthy control individuals at a 1:1 ratio to provide a physiological comparison of the postprandial response to the meal stimulus.

#### Data scale transformation

As a residual variance stabilising strategy***,*** the baseline and postprandial primary and secondary outcome data were logarithmically rescaled so that the pre- to postprandial outcome change represented the difference between log_*e*_(baseline outcome) and log_*e*_(postprandial outcome).

#### Analytical approach

For each outcome variable, two linear mixed model (LMM)-derived estimates for mean pre- to postprandial log_*e*_(outcome) were obtained for each protocol in the type 1 diabetes cohort. Similarly, an LMM-derived estimate for mean pre- to postprandial log_*e*_(outcome) change was obtained for each outcome in the control protocol.

#### Hypothesis testing

Two sets of null hypotheses were tested: one determined whether the mean pre- to postprandial log_*e*_(outcome) change was equal to zero within each protocol, and another determined whether the within-admission effect of prandial insulin timing on each outcome was the same between the two admissions of the type 1 diabetes crossover protocol and/or whether the within-admission effect was the same in the control protocol compared with each of the type 1 diabetes protocols.

#### Baseline outcome adjustment

In the framework of LMM analysis of covariance (ANCOVA), the between-protocol comparison of mean log_*e*_(outcome) change between the two type 1 diabetes protocols was covariate adjusted for log_*e*_(preprandial outcome). Similarly, the between-protocol comparisons of mean log_*e*_(outcome) change between each type 1 diabetes protocol comparison with the control protocol were covariate adjusted for preprandial log_*e*_(outcome).

#### Analytical tests

Baseline biochemical and vital sign characteristics of the two cohorts were compared with two-sample Student's *t* tests or two-sample Welch's *t* tests depending on whether variances of the outcome distributions were comparable or incomparable, respectively. For each protocol in the type 1 diabetes crossover, an LMM-derived paired *t* test was used to determine whether the mean within-protocol change was equal to zero and/or whether the mean within-protocol change was the same between protocols. In the control protocol, a conventional paired *t* test was used to determine whether the mean within-protocol change was equal to zero and a two-sample Welch's test was used to determine whether the within-protocol change was equal to changes seen within either of the type 1 diabetes protocols. Any between-protocol comparisons were baseline-adjusted for the specific variable being evaluated.

#### False discovery

Since each of the aforementioned sets of null hypotheses required three hypothesis tests, the Benjamini–Hochberg procedure [27] was applied to *p* values for each secondary outcome null hypothesis to maintain an overall false discovery error rate of 0.05.

#### Interpretation

Estimates of the pre- to postprandial mean log_*e*_(outcome) change and for the difference in pre- to postprandial mean log_*e*_(outcome) change were exponentiated to the geometric mean ratio scale so that effect size could be presented on a relative scale.

#### Missing data

We obtained complete datasets for all measured outcomes reported below and thus there were no missing data.

#### Computing methods

All analyses were conducted using SAS version 9.4 TS1M8 (SAS Institute; Cary, NC).

## Results

### Participant characteristics

We enrolled and randomised 18 people with type 1 diabetes and 18 age-, sex- and BMI-matched control participants. The two cohorts differed only by fasting blood glucose and HbA_1c_ values at baseline (Table 1). Each cohort had normal mean BMI and normotensive mean BP. In the type 1 diabetes cohort, mean urine albumin/creatinine was normal and mean C-peptide was <0.07 nmol/l (<0.2 ng/ml). Information on at-home insulin delivery methods, usual insulin:carbohydrate ratio and amount of prandial insulin administered during the current study to each participant with type 1 diabetes can be found in ESM Table 3. To align with recommendations on reporting menstrual cycle phase in women undergoing FMD assessment [22], we obtained serum progesterone values on each study date for all female participants and defined the post-ovulatory/luteal phase as progesterone ≥15.9 nmol/l (≥5 ng/ml) [28]. Notably, all seven women with type 1 diabetes met this definition (and thus were in the post-ovulatory/luteal phase) on each admission of the crossover protocol. Conversely, 5/7 female control participants met this threshold on their study date.

**Table 1:** Baseline characteristics of study participants

Characteristic	T1D cohort (*n*=18)	Control cohort (*n*=18)	*p* value
Sex (male; female)	11; 7	11; 7	N/A
Age (years)	25.7±4.3	25.2±4.3	0.70
Diabetes duration (years)	14±6.1	N/A	N/A
SBP (mmHg)	124.1±9.2	121.2±9.0	0.34
DBP (mmHg)	72.6±7.0	70.1±7.3	0.30
BMI (kg/m^2^)	24.4±3.4	24.1±2.0	0.72
FBG (mmol/l)	7.4±1.90	4.8±0.19	<0.001***
HbA_1c_ (mmol/mol)	53.1±10.0	32.5±3.0	<0.001***
HbA_1c_ (%)	7.0±0.9	5.1±0.3	<0.001***
Total-C (mmol/l)	4.2±0.7	4.5±0.8	0.36
LDL-C (mmol/l)	2.5±0.5	2.7±0.6	0.54
HDL-C (mmol/l)	1.4±0.3	1.5±0.3	0.62
TG (mmol/l)	0.7±0.2	0.9±0.5	0.26
C-peptide (nmol/l)	0.03±0.03	N/A	N/A
UAC (mg/mmol)	0.8±1.5	N/A	N/A

Data are presented as mean ± SD

^***^p<0.001

FBG, fasting blood glucose; HDL-C, HDL-cholesterol; LDL-C, LDL-cholesterol; N/A, not applicable; T1D, type 1 diabetes; TG, triglycerides; Total-C, total cholesterol; UAC, urine albumin/creatinine

### Plasma glucose and insulin

**Fig. 2:**
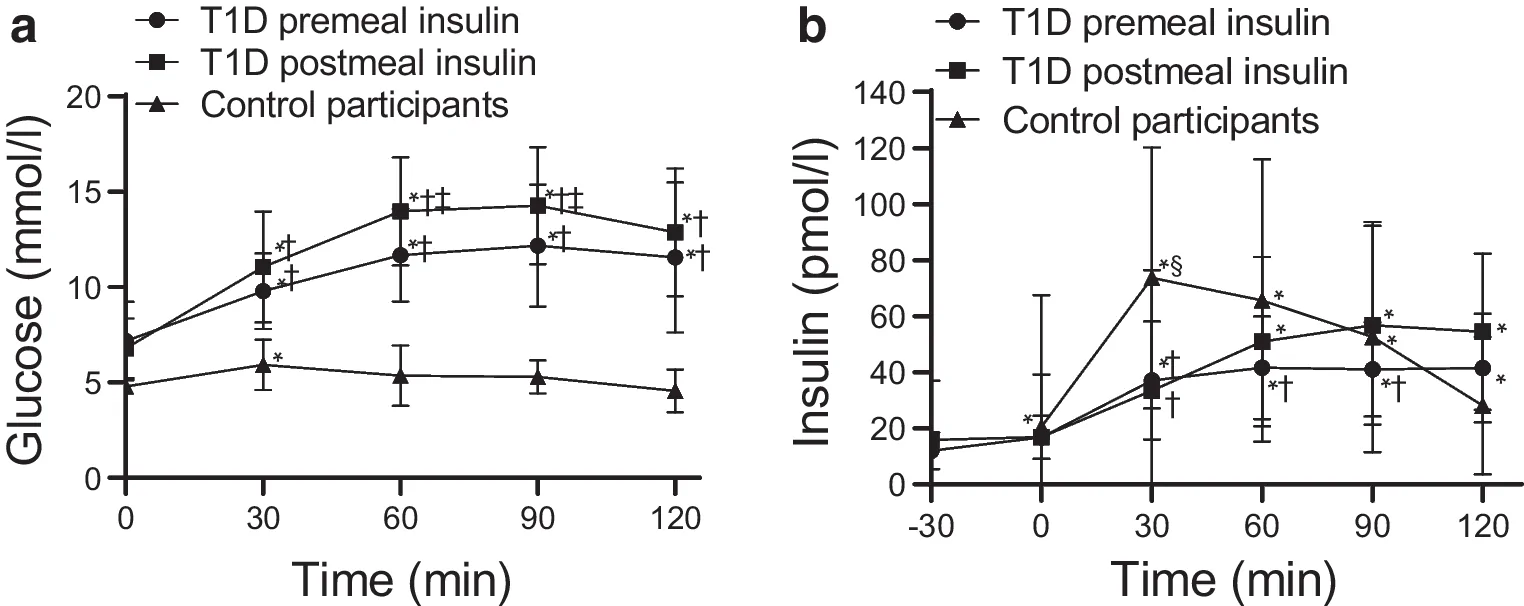
Plasma glucose (**a**) and insulin (**b**) concentrations over time in each study protocol. T1D, type 1 diabetes. *p<0.01 compared with protocol baseline; ^†^*p*<0.05 compared with control participant protocol; ^‡^*p*<0.05 compared with T1D premeal protocol; ^§^*p*<0.05 compared with either T1D protocol. Data are presented as mean ± SD error bars

Figure 2 demonstrates plasma glucose (Fig. 2a) and insulin concentrations (Fig. 2b) observed over time within each protocol. Notably, glucose significantly increased from baseline at every 30 min interval in each of the type 1 diabetes protocols. Conversely, change from baseline glucose was significant only at the 30 min timepoint in the control cohort. Comparing across protocols, plasma glucose was significantly higher at each timepoint in the two type 1 diabetes protocols when individually compared with the control cohort. Finally, plasma glucose was significantly higher at the 30, 60 and 90 min marks in the type 1 diabetes postmeal insulin admission compared with the premeal insulin admission. Peak mean plasma glucose occurred at the 90 min timepoint in each protocol and was 14.3 mmol/l (257.2 mg/dl) in the postmeal insulin protocol vs 12.2 mmol/l (219.4 mg/dl) in the premeal insulin protocol. Of note, 7/18 participants in the premeal insulin administration protocol achieved the guideline-recommended 2 h postprandial glucose target of <10.0 mmol/l (180 mg/dl) [14] while only 4/18 participants did so in the postmeal insulin administration protocol. Regarding geometric mean glucose area under the curve (AUC), each type 1 diabetes protocol demonstrated greater glucose AUC compared with the control protocol. Importantly, geometric mean glucose AUC was significantly greater (26,006 vs 22,685; *p*=0.015) in the postmeal insulin compared with the premeal insulin protocol in participants with type 1 diabetes.

Changes in plasma insulin concentration were significant at every 30 min interval in the type 1 diabetes premeal insulin protocol (Fig. 2b). Conversely, the change was not significant at the 0 min or 30 min mark in the type 1 diabetes postmeal insulin protocol but was significant at the 60, 90 and 120 min marks, suggesting that insulin began to exert earlier effects when injected before meal intake began. In the control group, change in plasma insulin was significant at the 30, 60 and 90 min marks but not the 120 min mark, suggesting a more rapid return to baseline values with endogenous than exogenous insulin. Mean change in plasma insulin was significantly greater at the 30 min mark in control participants compared with either type 1 diabetes protocol and was also significantly lower at the 120 min mark when compared with the type 1 diabetes postmeal protocol. Geometric mean insulin AUC did not significantly differ amongst any of the protocols.

### Primary outcome: myocardial MBF

**Fig. 3:**
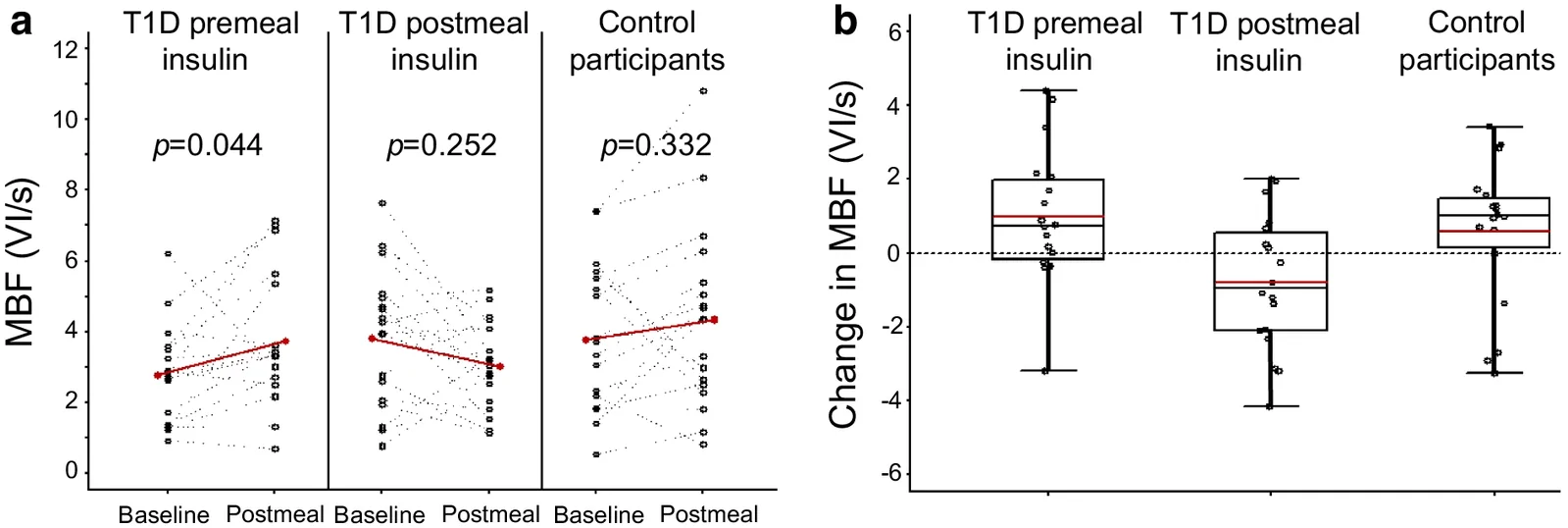
(**a**) Participant-specific pre- and postmeal MBF values. Black hatched lines identify the participant-specific MBF trajectories and red solid lines identify the group mean MBF trajectories. (**b**) Empirical distribution box plots for pre- to postmeal change in MBF within each protocol. Black interior-box horizontal lines identify the median of the MBF distribution and red interior-box horizontal lines identify the mean of the MBF distribution. Box plots represent the first and third quartile and whiskers represent the minimum and maximum values for each dataset. T1D, type 1 diabetes

Figure 3 and Table 2 demonstrate changes in myocardial MBV, MFV and MBF within each protocol. There were no changes (i.e. *p*>0.05) observed in MBV, MFV or MBF during either the control protocol or the postmeal insulin protocol in people with type 1 diabetes. However, MFV significantly increased (*p*=0.031) with premeal insulin administration in people with type 1 diabetes and this resulted in greater myocardial MBF (*p*=0.044).

**Table 2:** Fold change (postmeal:baseline) in geometric mean values for each myocardial variable broken down by cohort and protocol

Variable	Fold change in geometric mean	Lower 95% CI	Upper 95% CI	*p* value
MBV (VI)				
T1D premeal insulin	1.073	0.942	1.223	0.278
T1D postmeal insulin	0.886	0.778	1.010	0.068
Control participants	1.025	0.872	1.205	0.753
MFV (s)				
T1D premeal insulin	1.242	1.021	1.512	0.031*
T1D postmeal insulin	0.961	0.790	1.170	0.683
Control participants	1.125	0.942	1.344	0.180
MBF (VI/s)				
T1D premeal insulin	1.333	1.008	1.764	0.044*
T1D postmeal insulin	0.852	0.644	1.127	0.252
Control participants	1.153	0.854	1.557	0.332

^*^p<0.05

T1D, type 1 diabetes

### Secondary outcomes

There was a significant decline in SBP and DBP within each protocol; however, there were no significant changes in HR or RPP within any protocol or cohort (Table 3). These results suggest that the postprandial state exerted similar haemodynamic trends across all protocols and cohorts.

**Table 3:** Fold change (postmeal:baseline) in geometric mean values for each secondary outcome broken down by cohort and protocol

Parameter	Fold change in GM (postmeal:premeal)	Lower 95% CI	Upper 95% CI	*p* value	B–H significance threshold
Haemodynamics					
SBP (mmHg)					
T1D premeal insulin	0.956	0.923	0.991	0.016*	0.033
T1D postmeal insulin	0.955	0.922	0.990	0.014*	0.017
Control participants	0.948	0.906	0.993	0.026*	0.050
DBP (mmHg)					
T1D premeal insulin	0.930	0.880	0.980	0.015*	0.050
T1D postmeal insulin	0.920	0.860	0.970	0.005**	0.033
Control participants	0.900	0.850	0.960	0.002**	0.017
HR (bpm)					
T1D premeal insulin	1.053	0.996	1.113	0.066	0.033
T1D postmeal insulin	1.059	1.002	1.119	0.043	0.017
Control participants	1.001	0.956	1.049	0.948	0.050
RPP					
T1D premeal insulin	1.007	0.939	1.080	0.841	0.050
T1D postmeal insulin	1.012	0.943	1.085	0.740	0.033
Control participants	0.950	0.877	1.029	0.190	0.017
Aortic stiffness					
cfPWV (m/s)					
T1D premeal insulin	1.01	0.94	1.08	0.839	0.033
T1D postmeal insulin	0.97	0.91	1.04	0.384	0.017
Control participants	1.01	0.94	1.07	0.861	0.050
Conduit artery endothelial function					
Mean BA diameter (mm)					
T1D premeal insulin	1.018	0.994	1.042	0.137	0.017
T1D postmeal insulin	1.002	0.979	1.026	0.855	0.050
Control participants	1.005	0.984	1.026	0.628	0.033
Peak BA diameter (mm)					
T1D premeal insulin	1.019	0.997	1.042	0.087	0.017
T1D postmeal insulin	1.002	0.981	1.025	0.823	0.033
Control participants	1.001	0.982	1.021	0.888	0.050
FMD (%)					
T1D premeal insulin	1.018	0.994	1.042	0.137	0.017
T1D postmeal insulin	1.002	0.979	1.026	0.855	0.050
Control participants	1.005	0.984	1.026	0.628	0.033
Peak shear (s^−1^)					
T1D premeal insulin	1.059	0.918	1.222	0.420	0.050
T1D postmeal insulin	1.083	0.939	1.250	0.265	0.033
Control participants	1.212	1.081	1.360	0.003**	0.017
Time-to-peak diameter (s)					
T1D premeal insulin	1.695	0.623	4.617	0.291	0.033
T1D postmeal insulin	1.429	0.525	3.890	0.474	0.050
Control participants	5.646	0.972	32.803	0.053	0.017
Conduit artery flow					
Mean BA velocity (mm/s)					
T1D premeal insulin	1.737	1.284	2.349	0.001**	0.017
T1D postmeal insulin	1.569	1.160	2.122	0.005**	0.050
Control participants	1.583	1.216	2.060	0.002**	0.033
Peak PCD velocity (mm/s)					
T1D premeal insulin	1.080	0.938	1.242	0.275	0.050
T1D postmeal insulin	1.086	0.944	1.249	0.241	0.033
Control participants	1.214	1.080	1.364	0.003**	0.017
Skeletal muscle microvascular function					
MBV (VI)					
T1D premeal insulin	0.963	0.815	1.137	0.644	0.050
T1D postmeal insulin	0.846	0.717	0.999	0.049	0.017
Control participants	1.103	0.952	1.277	0.179	0.033
MFV (s)					
T1D premeal insulin	0.928	0.746	1.153	0.489	0.050
T1D postmeal insulin	0.864	0.695	1.074	0.181	0.017
Control participants	1.127	0.892	1.423	0.296	0.033
MBF (VI/s)					
T1D premeal insulin	0.974	0.687	1.381	0.881	0.050
T1D postmeal insulin	0.731	0.516	1.037	0.077	0.017
Control participants	1.242	0.873	1.769	0.212	0.033

^*^p<0.05, **p<0.01

BA, brachial artery; B–H, Benjamini–Hochberg; GM, geometric mean; PCD, photon-counting detector

Similarly, NEFA significantly decreased (*p*<0.001) within each protocol but this change did not differ between protocols (ESM Table 4). Conversely, there were no significant changes seen in CRP, IL-6, TNF-α, ICAM-1 or E-selectin in any protocol or cohort (ESM Table 4). There were also no significant changes in cfPWV observed within any protocol or cohort (Table 3), indicating that neither insulin timing nor the postprandial state in general influenced aortic stiffness. Similarly, there were no significant changes observed in mean or maximum brachial artery diameter or FMD in any protocol or cohort (Table 3), indicating that the investigational variables did not acutely alter conduit artery endothelial function. Of interest, E-selectin is a biomarker of endothelial dysfunction and the lack of change in this variable aligns with the lack of change seen in brachial artery FMD.

While no changes were observed in FMD, there were changes noted in brachial artery blood flow. Specifically, mean brachial artery flow velocity significantly increased within each protocol (Table 3). Notably, change in peak post-cuff-deflation velocity and peak shear rate were significantly greater in the control cohort but not in either of the type 1 diabetes protocols (Table 3). Summary statistics for pre- and postmeal FMD are presented in ESM Table 5. In skeletal muscle microvasculature, none of the changes observed in MBV, MFV or MBF met the Benjamini–Hochberg threshold for significance in any cohort or protocol (Table 3), indicating that neither insulin timing nor the postprandial state in general altered this endpoint.

## Discussion

While numerous studies have evaluated the effect of intravenous insulin on cardiovascular function, few have evaluated the effects of subcutaneous prandial insulin on this endpoint. Our group has previously reported that insulin (at physiologically relevant concentrations in the context of a euglycaemic insulin clamp) increased myocardial MBF in healthy humans [29], but the physiology of intravenous glucose and insulin do not necessarily match that of exogenous insulin and oral glucose [30]. To our knowledge, the current study is the first to evaluate the impact of specific prandial insulin timing strategies on such endpoints in people with type 1 diabetes. Most studies in this realm have focused on glucocentric outcomes, with the majority demonstrating that premeal insulin administration (ideally ~15–20 min before meal intake begins) leads to lower postprandial plasma glucose values [12, 31–34]. The only prior cardiovascular study using subcutaneous insulin therapy was a randomised, three-way, placebo-controlled crossover clinical trial by Scognamiglio et al that tested the effects of a saline injection immediately before meal intake vs insulin aspart injected immediately before meal intake vs regular insulin injected 25 min before meal intake in 20 control individuals and 20 people with type 2 diabetes [26]. Their results showed that glucose AUC was significantly lower with insulin aspart and this corresponded to increased postprandial MBF as measured by myocardial contrast echocardiography 120 min after meal intake began. In the same study, myocardial MBF decreased with placebo and regular insulin administration [26]. Here it should be noted that the peak action of regular insulin occurs ~2–4 h after injection [35] and thus participants in that study may not have seen full effect at the time when myocardial contrast echocardiography was performed. Despite the differing insulin administration strategies and study populations, our results mirror theirs by demonstrating that blunting postprandial hyperglycaemia with appropriately timed prandial insulin corresponds to greater myocardial MBF in people with type 1 diabetes.

Interestingly, the change in myocardial MBF in the current study was driven by an increase in MFV with no change in MBV. This finding aligns with recent murine studies showing that contrast-enhanced ultrasound is most effective at measuring changes in regional capillary and/or arteriolar flow velocity during insulin administration [36, 37]. One possible explanation for the observed change in MFV is that preprandial insulin injection leads to earlier stimulation of GLUT-4 translocation in cardiomyocytes that in turn improves myocardial glucose uptake (MGU) in the postprandial state [38]. In this way, the myocardium is primed for efficient MGU and the increased MFV reflects improved metabolic supply–demand matching. Insulin directly regulates MGU, particularly in the postprandial state wherein it promotes glucose oxidation [39]. Animal [40] and human [41–43] studies have repeatedly shown that acute insulin therapy (either via intraperitoneal or intravenous administration in mice or humans, respectively) can rescue MGU in type 1 diabetes. These changes in insulin-stimulated MGU then contribute to better myocardial metabolism and function, which in turn benefit myocardial MBF [44]. Furthermore, this relationship is probably bidirectional given that MGU is at least partially dependent on MBF [45]. Taken together, the changes we observed in MFV and MBF with preprandial insulin suggest that appropriately timed prandial insulin may lead to better temporal matching between substrate delivery and perfusion (i.e. metabolic–perfusion coupling). In this way, delayed insulin administration would cause nutrients to arrive first and the vascular effects of insulin to lag behind, resulting in a coupling mismatch where the peak metabolic demand window may have passed before the myocardial actions of insulin are fully realised. While these are plausible explanations, our study was not designed to answer these particular questions and did not assess MGU or myocardial metabolism, thus they remain opportunities for future investigation.

Some studies have reported that elevated resting MBF is associated with increased cardiovascular risk in the general population [46, 47]. However, to our knowledge this has not been studied in people with type 1 diabetes. A key distinction here, though, is that most studies assessing resting MBF as a predictor of adverse cardiovascular outcomes did so in the setting of true rest and not in the postprandial period. Here we assert that postprandial MBF is quite different from resting MBF and, while not a classic physiological stressor, the postprandial state does carry some physiological ramifications that differ greatly from the resting state. For example, in the current study there was a significant decrease in both SBP and DBP in the postprandial period of each cohort studied. Furthermore, a slight increase in MBF after a meal is physiologically expected (although the pattern and context of that increase matter much more than the direction alone). After meal consumption in healthy individuals, insulin levels rise and then cardiac work and metabolic demand increase, with MBF likewise modestly increasing. In people with type 1 diabetes, several factors distort this response (e.g. timing of prandial insulin administration, insulin sensitivity/absorption, etc.) and investigating this in detail was our rationale for the current study. Our results showed that administering prandial insulin 15 min before meal intake began led to reduced postprandial glucose and increased MBF, which would be the expected response based on substrate delivery and metabolic demand. Similarly, a late administration of insulin would be expected to alter that efficiency by leading to greater serum glucose and unchanged or lowered MBF, as our results demonstrated.

A larger clinical question that remains unknown, but warrants further investigation, is whether the lack of a myocardial MBF response seen with postmeal insulin administration in the current study either contributes to the development of myocardial microvascular dysfunction in people with type 1 diabetes or precipitates microvascular ischaemia in people with pre-existing microvascular disease. If true, this would highlight the value of appropriate premeal insulin timing as a method to potentially improve both gluco- and cardiometabolic health in this patient population. Of note, this ‘metabolic–perfusion uncoupling’ phenomenon carries downstream remodelling effects that have been linked to the development of diabetic cardiomyopathy [48]. This is especially important given that people with type 1 diabetes experience postprandial hyperglycaemia frequently [12, 49], and it is well-recognised that the current closed-loop AID systems lag in this domain [50], emphasising its relevance as a possible target for therapeutic intervention.

Regarding secondary outcomes, we observed no acute changes in HR, RPP, systemic inflammation, aortic stiffness (i.e. cfPWV), conduit artery endothelial function (i.e. brachial FMD) or skeletal muscle MBF within any protocol tested. While we were unable to measure changes in microvascular endothelial function or microvascular inflammation directly, the lack of change in systemic inflammation coupled with a similar lack of change in blood biomarkers of endothelial dysfunction and FMD itself suggests that these variables probably do not explain our observed changes in myocardial MBF. Conversely, we did observe changes in brachial artery flow velocity within each protocol. This result adds further credence to the idea that insulin and/or the postprandial state augment vascular flow velocity in various tissues [36, 37, 51]. We also observed significant increases in peak post-cuff-deflation velocity and peak shear rate in control participants but not in the type 1 diabetes protocols. While FMD itself did not change in any protocol, the changes in peak shear and post-cuff-deflation velocity occurring only in control participants suggest that at least some components of the brachial artery vascular response to meal intake differ between the two populations. Concerning the lack of change in inflammation, we note that most data showing acute changes in inflammatory biomarkers were reported in the context of an intravenous insulin clamp [52, 53] as opposed to subcutaneous insulin injection in people with type 1 diabetes. However, the SIT-LESS randomised crossover clinical trial evaluated the impact of physical activity after a meal (vs remaining sedentary) on postprandial cardiovascular markers in 32 people with type 1 diabetes. In this study, participants used the same prandial insulin strategy in the crossover schema (thus ensuring that physical activity was the only altered variable between protocols) and inflammatory biomarkers were measured 3.5 and 7 h after meal intake. Notably, postprandial levels of TNF-α significantly increased in the sedentary protocol while they did not significantly change in the light-intensity physical activity arm. Our participants were sedentary in the postprandial observational period, so the lack of change we observed could be due to the shorter monitoring period in our study vs theirs (i.e. 2 h vs 3.5 h) or lack of statistical power.

Our study has several strengths that should be highlighted, including the rigorous methodology, novelty of the investigated question and ability to evaluate both biochemical and functional vascular outcomes. However, despite these strengths, there are several limitations that also warrant discussion. First, as previously mentioned, we were unable to measure MGU. While measuring MGU would not be expected to alter our primary finding of increased myocardial MBF, it would provide useful mechanistic insight into the concurrent physiology and its impact on myocardial energetics. Second, our type 1 diabetes cohort was young, had good glycaemic control at baseline and lacked microvascular complications. Future studies are needed to determine whether similar results are observed in older cohorts with microvascular complications and/or younger cohorts with poor baseline glycaemic control. Third, our methodology allows us to mark the ultrasound probe location within studies but not between studies. Thus, we were unable to provide across-protocol comparisons of myocardial or skeletal MBF. There were baseline differences in myocardial MBF between cohorts and, while this is a known limitation of myocardial contrast echocardiography, it should be taken into account when interpreting our results. Fourth, we used contrast-enhanced ultrasound as the imaging methodology of choice because our group has extensive experience with it. However, we note that positron emission tomography (PET) with oxygen-labelled water is considered the noninvasive gold standard for MBF measurement [54]. Fifth, some studies have shown that acute hyperglycaemia (in the context of a hyperglycaemic insulin clamp) has a deleterious effect specifically on myocardial vasodilator function [55]. We were unable to administer a vasodilator agent and assess myocardial perfusion reserve in the current study, and thus this remains a question for future investigation. Similarly, we were unable to administer nitroglycerin and determine endothelium-independent vasodilatation in the brachial artery. This was due to the fact that administering nitroglycerin would have interfered with subsequent determination of myocardial MBF within our protocol. Sixth, plasma insulin levels saw an earlier increase in the premeal insulin protocol but the AUC for insulin did not differ between the type 1 diabetes protocols. This finding generally aligns with the idea that myocardial metabolic and vascular responses depend not only on the insulin dose administered, but also on the temporal relationship between numerous factors such as plasma insulin, glucose appearance, nutrient delivery, etc. that each contribute to appropriate metabolic–perfusion coupling. However, we note that we were unable to completely delineate whether the observed changes in myocardial MBF in the current study were specifically due to reduced hyperglycaemia, earlier insulin administration or some combination of each. We favour the latter interpretation, but this remains a question for future investigation. Seventh, the small sample size precluded our ability to conduct analyses by sex. It will be important to determine whether the effects identified in the current study differ by sex in future studies. Finally, we note that the majority (11/18 participants) of our type 1 diabetes premeal insulin administration group did not reach the guideline-recommended 2 h postprandial glucose target of <10.0 mmol/l (180 mg/dl) [14]. It would be valuable for future studies to determine whether there is any additional cardiovascular benefit when hitting this specific glucose threshold.

### Conclusions

In this single-centre, randomised, crossover clinical trial, we found that appropriately timed premeal insulin led to lower postprandial blood glucose along with increased myocardial MBF in people with type 1 diabetes. Further work is needed to determine the underlying physiology of these changes.

## Supplementary Information

Below is the link to the electronic supplementary material.

ESM (PDF 434 KB)

## Data Availability

The datasets generated and analysed during the current study are stored in a password-protected electronic database. Access to these datasets is available immediately from the corresponding author upon reasonable request. Access will include a unique password that provides user access to the datasets. Those seeking access will be granted a 30 day period wherein they may analyse primary and secondary outcome data.

## Abbreviations

AID: Automated insulin delivery
AUC: Area under the curve
BP: Blood pressure
cfPWV: Carotid–femoral pulse wave velocity
CONSORT: Consolidated Standards of Reporting Trials
CRP: C-reactive protein
CRU: Clinical Research Unit
DBP: Diastolic blood pressure
FMD: Flow-mediated dilation
HR: Heart rate
ICAM-1: Intercellular adhesion molecule-1
IL-6: Interleukin-6
LMM: Linear mixed model
MBF: Microvascular blood flow
MBV: Microvascular blood volume
MFV: Microvascular flow velocity
MGU: Myocardial glucose uptake
MI: Mechanical index
NEFA: Non-esterified fatty acids
RPP: Rate pressure product
SBP: Systolic blood pressure
TNF-ɑ: Tumour necrosis factor-ɑ
UVA: University of Virginia
VI: Video intensity
